# Understanding load in netball – An analysis of multiple seasons, phases, and teams

**DOI:** 10.1371/journal.pone.0266830

**Published:** 2022-04-13

**Authors:** Lyndell Bruce, Tanisha Bardzinski, Dan Dwyer

**Affiliations:** Centre for Sport Research, School of Exercise and Nutrition Sciences, Deakin University, Burwood, Victoria, Australia; Universidade Federal de Mato Grosso do Sul, BRAZIL

## Abstract

Studies of training and competition load in sport are usually based on data that represents a sample of a league and or annual training program. These studies sometimes explore important factors that are affected by load, such as training adaptations and injury risk. The generalisability of the conclusions of these studies, can depend on how much load varies between seasons, training phases and teams. The interpretation of previous load studies and the design of future load studies should be influenced by an understanding of how load can vary across seasons, training phases and between teams. The current study compared training loads (session rating of perceived exertion x session duration) between all (8) teams in an elite Netball competition for multiple (2) season phases and (2) seasons. A total of 29,545 records of athlete session training loads were included in the analysis. Linear mixed models identified differences between seasons and training phases (p < .05). There were also differences between teams and a complex set of interactions between these three factors (season, phase, and team) (p < .05). While the absolute value of the training loads reported here are only relevant to elite netball, these results illustrate that when data is sampled from a broader context, the range and variation in load may increase. This highlights the importance of cautiously interpreting and generalisation of findings from load studies that use limited data sets.

## Introduction

Practitioners monitor athlete training loads for several reasons including managing fatigue, ensuring players are adequately prepared for competition, identifying injury risk, and determining player readiness [1]. The way training load is measured and how athletes respond to the imposed training stimulus can vary significantly between teams and sports [2]. Two common classifications of training load are internal and external load measures [3]. External load is a measure of the load specific to the training undertaken and includes measures such as distance [total distance or distance within specific speed ranges], accelerations and tonnage for resistance training [4]. Internal loads reflect the actual psycho-physiological response by the athlete elicited by the external training load and includes measures related to heart rate (e.g., heart rate variability, heart rate reserve) and the product of session rating perceived exertion (sRPE) and duration (sRPE-TL (training load)) [4].

A pre-season phase is designed to prepare athletes for the demands on the upcoming season and would be expected to have higher training loads than in-season due to the recovery required post match [5]. Previous research has supported this theory showing that pre-season loads are higher than in-season loads [5–7]. Aside from differences in season phases, we would also expect to see differences in the training loads experienced by different teams within the same competition. Several reasons could contribute to potential differences; different coaches and support staff, diverse training schedules, varied priorities on training components to name a few. Whilst these differences could be expected, no previous research has demonstrated how different these training loads may be between teams and how this might be reflected overall in the competition profile. Understanding the extent of these differences may assist in interpreting the robustness of previous studies who have only examined one team and/or season phase.

A relatively large proportion of the existing studies of load, use data from a single team or club and may have used data from a single season. For example, all the studies included in two reviews on training load [2, 8] relied on data from one club or team. Depending on the nature of data collected and analyses being conducted, using athletes from one team only, may result in lack of power which is often not reported in the literature [2]. Furthermore, existing studies often only report data from one season phase (e.g., pre-season or in-season) or one season cycle, therefore we have a lack of understanding of how load changes over time.

**Fig 1:**
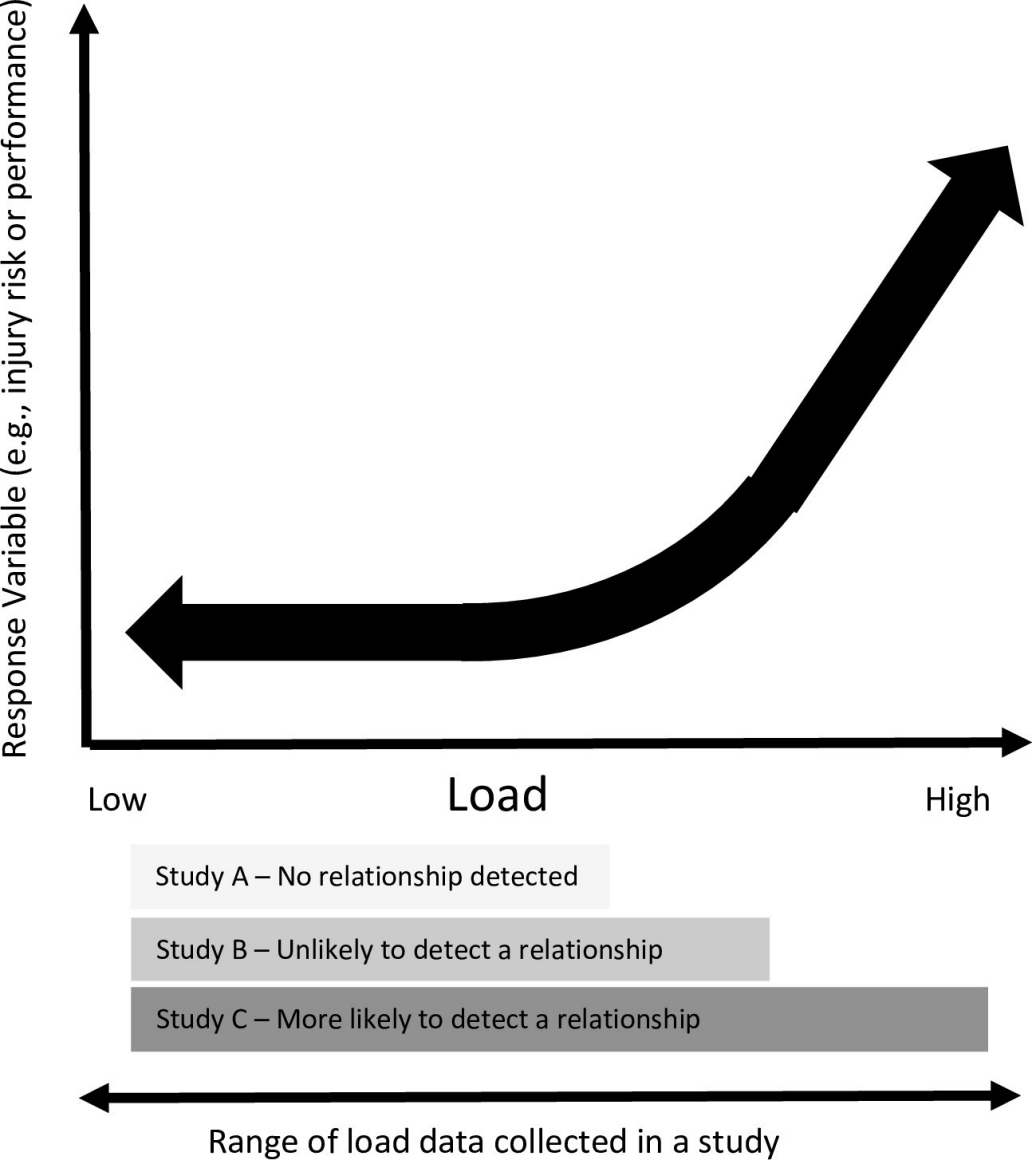
An example of the risks associated with analysing unrepresentative load data. This chart provides a conceptual illustration of a relationship between load and a response variable which could be a change in a component of fitness or injury risk. The horizontal bars represent the range of a load measure for three hypothetical studies. Each study’s unique range of load influences whether a relationship with the response variable would be detected in an analysis of the data.

A relatively small and or short-term sample of training load information has the potential to create other problems for researchers. There are many studies that seek to identify relationships between training load and important response variables such as, a change in a fitness component, injury risk or performance. We believe that there is a risk that the adequacy of the training load data used by these studies may affect their conclusions. This could occur when the training load data does not represent the complete range of training loads experienced by athletes in that sport. Fig 1 illustrates how data obtained from three different studies may lead to different conclusions. Data from the fictitious “Study A” may have been obtained in a season phase, under the influence of a conservative coach that represents the lower end of the complete range of training load that exists in the sport. Given the underlying relationship, the study would find no relationship between training load and their response variable. A different study based on data from “Study B”, may detect a weak relationship, whereas data from “Study C” may report a very strong relationship. None of these datasets alone capture the type of the underlying relationship, although data from only one study (i.e. Study A) is likely to avoid a false negative conclusion.

Previous studies of training load provide valuable information about typical training load in a variety of sports and have explored relationships between training load and other factors that affect performance, such as adaptations to training and injury risk. However, there is a need to explore the extent to which training load varies between teams, season phases and seasons. An understanding of these variations is required if we want to accurately quantify the typical range of training loads in sport. This understanding may also improve the likelihood that future studies are able to detect and identify relationships with other performance related factors. Collectively, this information may assist teams in determining effective and safe training loads for teams and athletes. Therefore, the aim of the current study was to examine the extent to which training load varies between all teams who compete in the same league, also between seasonal phases and between years. This study did not aim to explore relationships between training load and a response variable such as performance as we believe it is important to empirically demonstrate differences that potentially occur between teams, years, and season phases.

## Methods

Session rating of perceived exertion (sRPE-TL) was obtained from Netball Australia’s Athlete Management System (AMS) and contained player data from the first two seasons of Australia’s National Netball Competition (known as Suncorp Super Netball (SSN)). The competition includes eight teams each with 10 contracted players and up to five training partners. A total of 95 unique players provided sRPE-LT data over two seasons. The average age of the female athletes was 27.1 ± 4.2 years. Ethics approval was provided from the Australian Institute of Sport and Deakin University human research ethics committees.

Athletes from each team were asked to enter a rating of perceived exertion (RPE) after each session they completed, either training or match, as well as the duration of the session. Each athlete was required to enter their RPE via dropdown menu and session duration (free text entry) via a smartphone application and the entry included the date of the session. The sRPE-TL was automatically calculated from the session RPE and session duration (RPE x session duration in minutes).

After being downloaded from the AMS, data preparation was conducted. This included labelling all sessions (entries) by season phase (pre-season, in-season, finals, out of season), year (2017, 2018) and team (A–H). To enable the identification of season phases, each SSN team provided the start date of their pre-season for both 2017 and 2018, and the competition start end date was obtained from the SSN fixture for each season. An independent t-test revealed no significant difference in the average sRPE-TL per session between in-season and finals, so this data was combined for further analyses. Any data which was outside the pre- and in-season phases was excluded from the analysis as these were not stipulated team-based training sessions. Data entered when athletes were not involved in the SSN teams (i.e., international duties) were excluded from future analysis.

Potential outliers and errors in the data were identified and removed. The RPE and duration columns were examined, and any blank values in either column resulted in the removal of that data point, resulting in 112 entries being removed. Any session duration values over 180 minutes were further examined to determine if they were a valid session. This value was selected as it indicated a training session of greater than three hours and most reported netball training session lengths are less than this time [9, 10]. If a session was over 180 minutes, it was compared to other recorded sessions on the same date by athletes from the same team. If multiple entries were recovered over 180 minutes for the same day and team, the session was considered valid. Twenty-five entries were removed from the analysis as they were isolated sessions over 180 minutes. The final number of entries for analysis was 29,545.

### Statistical analysis

Teams did not complete the same number of training sessions therefore sRPE values were averaged across all sessions completed for each player rather than using weekly or other time-based measures. To remove interdependency between individual values, data were collapsed to a single median value for sRPE-TL, duration and RPE for each participant for each competition, year, season phase and session type. Normality of data was assumed given the relatively large sample size [11, 12]. To examine how individual teams may differ to competition wide variations in training loads, a linear mixed model (LMM) was run using all data (competition wide LMM) followed by separate LMM for each team. For the competition wide LMM, team was included as a random factor. For all LMM, year, season phase and the year x season phase interaction, were included as fixed factors. Alpha was set at p < .05. Data was analysed using Statistical Package for Social Sciences (Version 25.0, IBM Corp, Armonk, NY).

## Results

**Fig 2:**
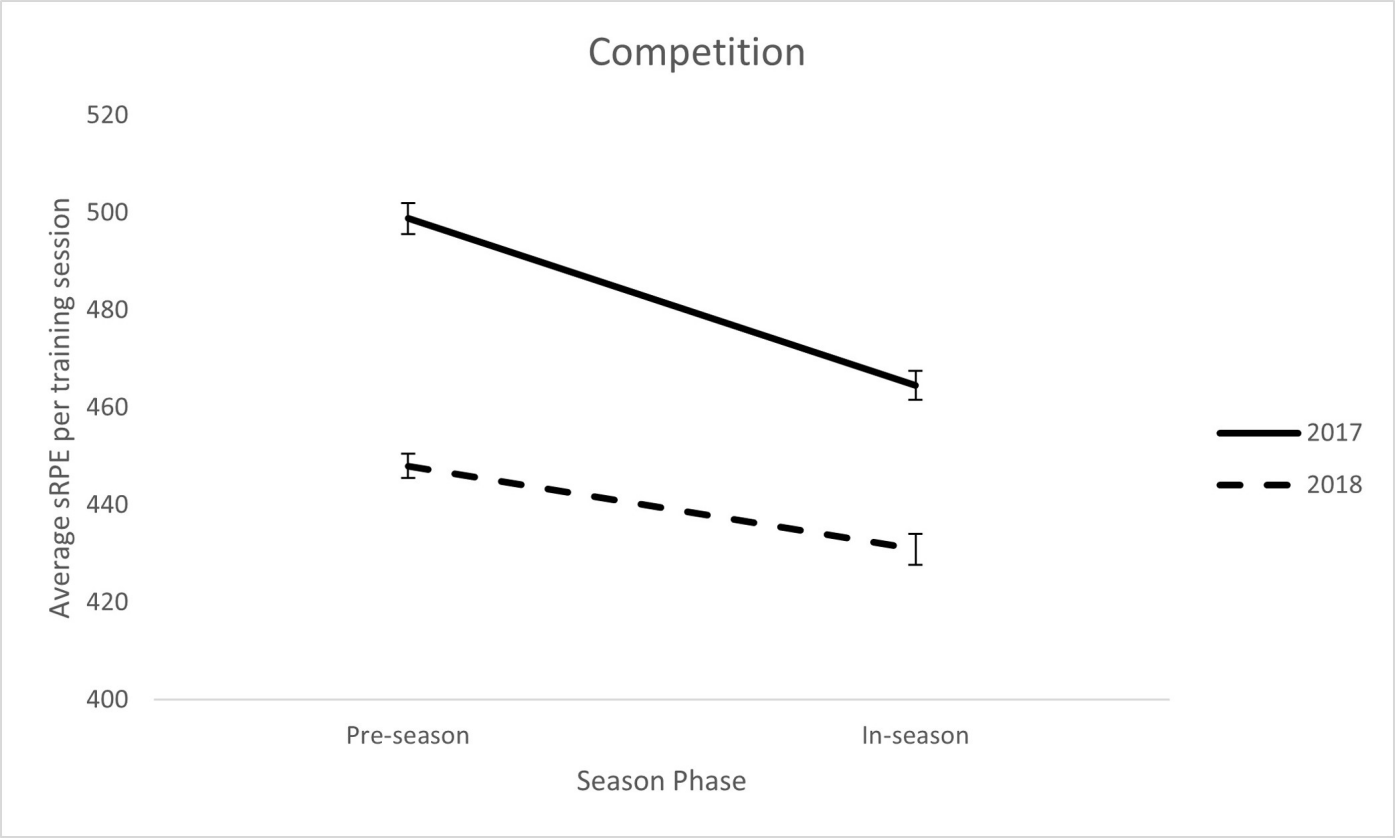
Average sRPE-TL for season phase and year across for all teams combined.

Results from the competition wide LMM analysis revealed significant main effects for year (F(1, 29538.39) = 227.97, p < .001) and season phase (F(1, 29535.93) = 94.67, p = < .001) and a significant year x season phase interaction (F(1,29534.58) = 10.48, p = .001) (see Fig 2). Training load was higher in pre- versus in-season, and training load was higher in 2017 compared with 2018.

Results from the analyses of each team are provided in Table 1. There was a difference in training load between 2017 and 2018 for four of the eight teams. There was a difference in load between pre- and in-season for five of the 8 teams. There was an interaction between these two factors (year and season phase) for five of the eight teams.

**Table 1:** Results of the linear mixed model for each individual team.

Team	df	Year main effect	Season phase main effect	Year x Season phase interaction
A	1, 4614	F = 1810, p < .001 ^#^	F = 2.04, p = .15	F = 12.23, p < .001
B	1, 3316	F = 0.001, p = .97	F = 0.69, p = .41	F = 6.30, p = .01
C	1, 3452	F = 0.68, p = .41	F = 112.77, p < .001 *	F = 29.54, p < .001
D	1, 3369	F = 29.96, p < .001 ^$^	F = 9.90, p = .002 ^	F = 0.14, p = .71
E	1, 2760	F = 7.10, p = .008 ^#^	F = 12.10, p = .001 *	F = 9.54, p = .002
F	1, 4031	F = 1.20, p = .27	F = 0.10, p = .76	F = 1.35, p = .25
G	1, 4650	F = 10.60, p = .001 ^#^	F = 8.09, p = .004 *	F = 10.01, p = .002
H	1, 3321	F = 3.69, p = .06	F = 56.44, p < .001 *	F = 1.80, p = .18

^#^ 2017 sRPE-TL greater than 2018 sRPE-TL.

^$^ 2018 sRPE-TL greater than 2017 sRPE-TL.

* Pre-season sRPE-TL greater than in-season sRPE-TL.

^ In-season sRPE-TL greater than pre-season sRPE-TL.

**Fig 3:**
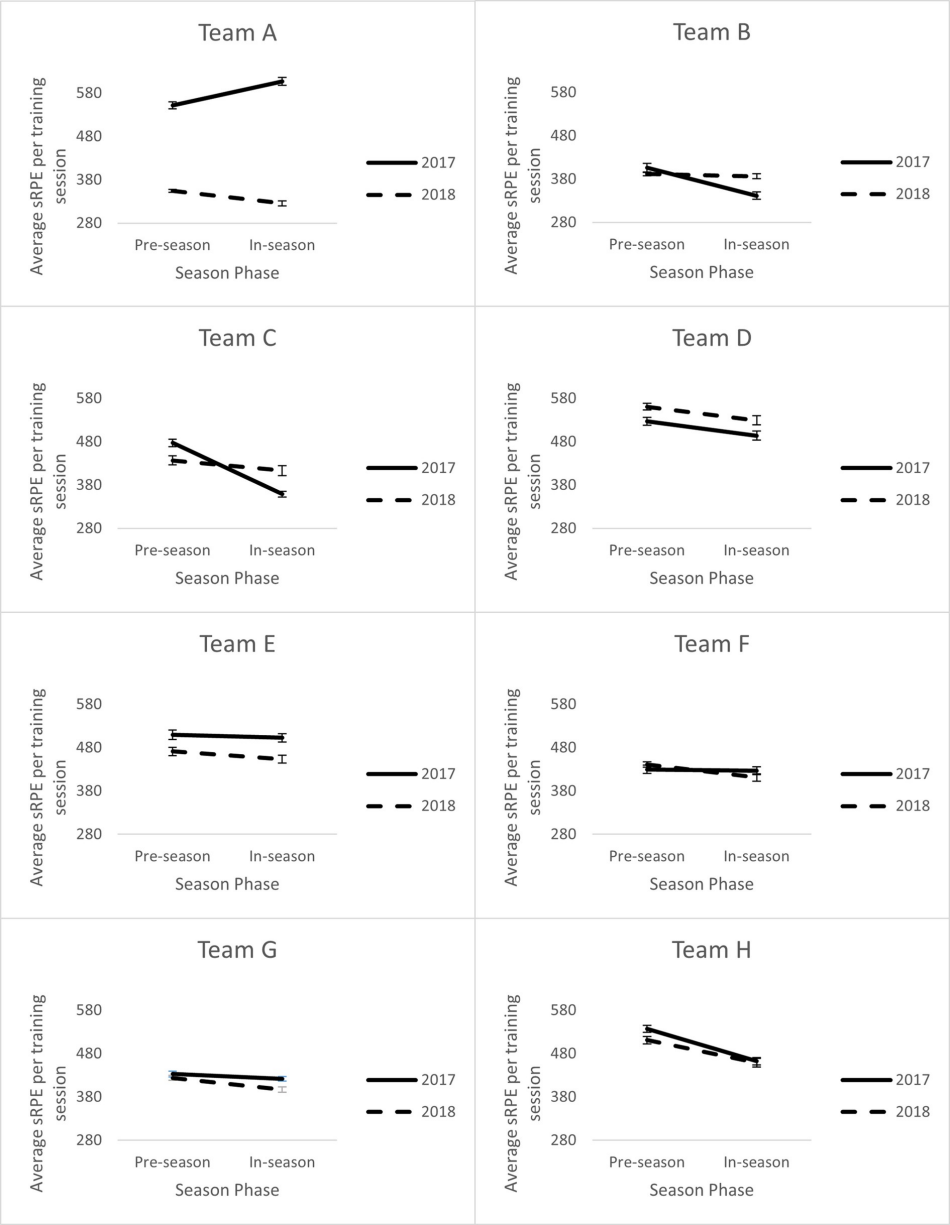
Year by season phase interaction for all teams.

Visualisations of the main effects of year and season phases on load are presented in Fig 3. The analysis of the main effects indicates that only some teams exhibited differences (changes) in training load between years and season phases. Moreover, where changes did occur, they were not always in the same direction. For example, of the four teams that exhibited a change in training load from 2017 to 2018, three declined and one increased. When examining the change in training load from pre- to in-season, four teams exhibited a decline, while one had an increase. Fig 3 also illustrates the variation in the interactions between year and season phase. It is evident that there are differences in the absolute training load of training sessions as well as variation in the direction of change in training load from 2017 to 2018 and from pre- to in-season. Only teams B and F did not exhibit a change in training load by year or by season phase, however a significant interaction was observed for B.

## Discussion

Our understanding of training load has been predominately limited to research examining one team within one year, thus we do not understand elite athlete’s training loads across multiple teams participating in the same competition, nor over multiple season phases and years. The current project has revealed significant differences not only exist between teams from the one season but also across season phases and years. This is not an unexpected finding, however, does suggest caution needs to be applied when generalising findings from one study to other teams, competition levels and across years. A discussion of the current findings is presented along with considerations for future research examining training loads in sport.

This is the first study to compare training loads across multiple years in a female court-based population. The average session load across the competition decreased in the 2018 season compared to the 2017 season. Whilst this trend was evident and significant differences were shown for three teams, one team demonstrated an increased training load in 2018. Two of the three teams which had the highest training loads in 2018 decreased their training loads in 2017 (teams A and E). Team E maintained their pre-season training load across both years but had a significant drop in their in-season training loads indicating a specific adjustment they felt needed to be made across the seasons. In contrast, Team A significantly decreased pre- and in-season training loads and showed the greatest adjustments of any team between 2017 and 2018. This indicates some specific adjustments to the way they prepared for matches in 2018. Previous research, albeit examining only one team has been mixed. Coppalle et al. [13] observed no difference in pre-season external and internal training loads for professional soccer players over two years. Whereas Gabbett [14] showed a reduction from one season to the following two seasons in sub-elite rugby league athletes. The varied nature of the current findings and previous research suggests practitioners differ in their approach to training load prescription. Future research should explore the relationship between changes in training load and individual and team performance.

Half of the teams in the competition had athletes complete more training load per session in the pre-season phase than in-season. Only one team (Team D) had higher training loads in-season than pre-season. Interestingly Team B had a significant year by season phase interaction, however, there were no main effects; in 2017, in-season training loads were lower than pre-season, however this was reversed for 2018. Multiple studies have reported higher pre-season training loads than in-season training loads [15–19] however, this is the first female study reported. Current findings, bar one team, are consistent with the notion that pre-season is typically used to prepare players for the physiological requirements of the sport in addition to technical and tactical preparation as the season draws closer [20]. A pre-season phase will typically have multiple macro and micro cycles, but the overarching plan is to prepare for the season ahead. One team is an apparent outlier (team D), which is interesting as it could be perceived that they are not preparing their athletes for the season ahead. In both years, players in Team D reported working harder in-season (via RPE) than pre-season with no difference in the length of sessions. Further understanding around individual and team performance in-season may provide further insights into whether this was a successful strategy for this team.

The competition trend was for higher training loads in pre-season than in-season and higher training loads in 2017 compared to 2018. Only two of the eight teams demonstrated the same main effects as the competition wide results. No teams had the same interaction effect as the competition wide results. It is not surprising that teams differed in the training load patterns to the competition wide results. This highlights how understanding training load across multiple teams within a competition may allow a team/s to identify if they are over- or under-training their athletes with respect to the remainder of the competition. They can then use this information in addition to performance metrics to assess whether this may present additional challenges or issues. Competition managers may also be able to play a role in notifying teams of over- or under-training if there is risk of athlete injury (either through under- or over-loading) or overtraining occurring. Having oversight on player loads across a competition may also be important if players are then selected into representative teams for ‘higher’ competitions (e.g., international matches, state of origin). Understanding a player’s more recent training loads could ensure appropriate loading (e.g., building or decreasing loads within an acceptable range) to enhance performance in the new environment.

Whilst the findings of the current study provide some interesting findings, the use of sRPE-TL is not without limitations [21]. It is recommended that further studies aim to include both objective and subjective measures of load, notwithstanding the barriers to implementing this across a competition (e.g., cost of devices, teams using the same hardware). Whilst we have addressed some factors in our analysis, we have not examined changes across different levels of competitions longitudinally and this should be a focus of future research. The current study used sRPE-TL as a measure of session load and teams may have completed different numbers of sessions within a week, thus the overall training load between teams may be vastly different. It could be speculated that some teams may have had lower session loads and completed more sessions in a week (e.g., Team B), whereas other teams may have had higher session loads and completed fewer sessions in a week (e.g., Team H). Future studies should look to examine weekly training loads and their relationship to competition preparation.

## Practical applications

Findings from the current study suggest that there is risk in inappropriately generalising findings about training load, from one team to a league or one season phase to a year or one year to another. Only one of the eight teams in the competition (Team F) did not show any differences between phases and years. All other teams demonstrated changes in the range of their training loads across the season phases or two years of the competition. Future studies may be limited in their capacity to be generalised if they do not implement careful research design. Single team designs may provide an indicator of normative training load values for a sport or competition level, however, as shown in the current study, may not be representative of all teams. Therefore, caution should be applied when using single team results as normative data.

## References

[1] Bourdon PC, Cardinale M, Murray A, Gastin P, Kellmann M, Varley MC, et al. Monitoring athlete training loads: Consensus statement. Int J Sports Physiol Perform. 2017;12(Suppl 2):S2-161-S2-170. doi: 10.1123/IJSPP.2017-0208 28463642

[2] Fox JL, Stanton R, Sargent C, Wintour S-A, Scanlan AT. The association between training load and performance in team sports: A systematic review. Sport Med. 2018;48:2743–74. doi: 10.1007/s40279-018-0982-5 30225537

[3] Impellizzeri FM, Rampinini E, Marcora SM. Physiological assessment of aerobic training in soccer. J Sports Sci. 2005;23(6):583–92. doi: 10.1080/02640410400021278 16195007

[4] Impellizzeri FM, Marcora SM, Coutts AJ. Internal and external training load: 15 years on. Int J Sports Physiol Perform. 2019;14(2):270–3. doi: 10.1123/ijspp.2018-0935 30614348

[5] Moreira A, Coutts AJ, Moreira A, Bilsborough JC, Sullivan CJ, Cianciosi M, et al. Training periodization of professional Australian football players during an entire Australian Football League season. Int J Sports Physiol Perform. 2015;10:566–71. doi: 10.1123/ijspp.2014-0326 25405365

[6] Aoki MS, Ronda LT, Marcelino PR, Drago G, Carling C, Bradley PS, et al. Monitoring training loads in professional basketball players engaged in a periodized training program. J Strength Cond Res. 2017;31(2):348–58. doi: 10.1519/JSC.0000000000001507 27243913

[7] Jeong TS, Reilly T, Morton J, Bae SW, Drust B. Quantification of the physiological loading of one week of “pre-season” and one week of “in-season” training in professional soccer players. J Sports Sci. 2011;29(11):1161–6. doi: 10.1080/02640414.2011.583671 21777053

[8] McLaren SJ, Macpherson TW, Coutts AJ, Hurst C, Spears IR, Weston M. The relationships between internal and external measures of training load and intensity in team sports: A meta-analysis. Sport Med. 2018;48(3):641–58. doi: 10.1007/s40279-017-0830-z 29288436

[9] Young CM, Gastin PB, Sanders N, Mackey L, Dwyer DB. Player load in elite netball: Match, training, and positional comparisons. Int J Sports Physiol Perform. 2016;11(8):1074–9. doi: 10.1123/ijspp.2015-0156 27001768

[10] Chandler PT, Pinder SJ, Curran JD, Gabbett TJ. Physical demands of training and competition in collegiate netball players. J Strength Cond Res. 2014;28(10):2732–7. doi: 10.1519/JSC.0000000000000486 24983848

[11] Glass G V, Peckham PD, Sanders JR. Consequences of failure to meet assumptions underlying the fixed effects analyses of variance and covariance. Rev Educ Res. 1972;42(3):237–88.

[12] McDonald JH. Handbook of biological statistics. 3rd ed. Baltimore, Maryland: Sparky House Publishing; 2014.

[13] Coppalle S, Rave G, Ben Abderrahman A, Ali A, Salhi I, Zouita S, et al. Relationship of pre-season training load with in-season biochemical markers, injuries and performance in professional soccer players. Front Physiol. 2019;10:409. doi: 10.3389/fphys.2019.00409 31031638 PMC6474299

[14] Gabbett TJ. Reductions in pre-season training loads reduce training injury rates in rugby league players. Br J Sports Med. 2004;38(6):743–9. doi: 10.1136/bjsm.2003.008391 15562171 PMC1725000

[15] Black CJ, Till K, O’Hara JP, Davidson J, Jones B. Top secret training data? External training loads of a cup winning English Super League rugby league team. Int J Sport Sci Coach. 2018;13(2):236–42.

[16] Dubois R, Paillard T, Mcgrath D, Chamari K, Maurelli O, Polly S, et al. Changes in training load, running performance, lower body power and biochemical characteristics of back players throughout a professional rugby union season. J Hum Sport Exerc. 2017;12(1):1–16.

[17] Granados C, Izquierdo M, Ibàñez J, Ruesta M, Gorostiaga EM. Effects of an entire season on physical fitness in elite female handball players. Med Sci Sports Exerc. 2008;40(2):351–61. doi: 10.1249/mss.0b013e31815b4905 18202565

[18] Malone JJ, Di Michele R, Morgans R, Burgess D, Morton JP, Drust B. Seasonal training-load quantification in elite English Premier League soccer players. Int J Sports Physiol Perform. 2015;10(4):489–97. doi: 10.1123/ijspp.2014-0352 25393111

[19] Ritchie D, Hopkins WG, Buchheit M, Cordy J, Bartlett JD. Quantification of training and competition load across a season in an elite Australian football club. Int J Sports Physiol Perform. 2016;11(4):474–9. doi: 10.1123/ijspp.2015-0294 26355304

[20] Bangsbo J. The physiology of soccer—With special reference to intense intermittent exercise. Acta Physiol Scand Suppl. 1994;151(619):1–155. 8059610

[21] Herman L, Foster C, Maher MA, Mikat RP, Porcari JP. Validity and reliability of the session RPE method for monitoring exercise training intensity. South African J Sport Med. 2006;18(1).

